# Complete Genome Sequence of Cupriavidus necator H16 (DSM 428)

**DOI:** 10.1128/MRA.00814-19

**Published:** 2019-09-12

**Authors:** Gareth T. Little, Muhammad Ehsaan, Christian Arenas-López, Kamran Jawed, Klaus Winzer, Katalin Kovacs, Nigel P. Minton

**Affiliations:** a Nottingham BBSRC/EPSRC Synthetic Biology Research Centre (SBRC), Centre for Biomolecular Sciences, University of Nottingham, Nottingham, United Kingdom; University of Maryland School of Medicine

## Abstract

The hydrogen-utilizing strain Cupriavidus necator H16 (DSM 428) was sequenced using a combination of PacBio and Illumina sequencing. Annotation of this strain reveals 6,543 protein-coding genes, 263 pseudogenes, 64 tRNA genes, and 15 rRNA genes.

## ANNOUNCEMENT

The betaproteobacterium Cupriavidus necator H16 is a Gram-negative, mesophilic, non-spore-forming, facultatively chemolithoautotrophic bacterium able to grow on organic substrates or with H_2_ and CO_2_ under aerobic conditions (1). With high metabolic versatility and an ability to store a large amount of organic carbon in the form of poly-3-hydroxybutyrate (2), this is the chosen chassis organism of the Synthetic Biology Research Centre at The University of Nottingham (SBRC-Nottingham) for sustainable production of chemicals and fuels from CO_2_. Before embarking on a metabolic engineering project, it is vital to resequence the wild-type strain to ensure that any subsequent genome changes accumulated during passaging are identified. Long-read sequencing allows for the resolution of complex repeat regions, which may have been incorrectly assembled using earlier technologies. Errors in the long-read sequencing are then corrected by short-read sequencing (3).

Cupriavidus necator H16 (DSM 428) was obtained from the DSMZ strain collection and cultivated in Luria-Bertani (LB) medium supplemented with 10 μg/ml gentamicin at 30°C and 200 rpm. DNA was extracted from the culture using a GenElute bacterial genomic DNA kit (catalog number NA2110-1KT; Sigma-Aldrich).

A 12-kbp SMRTBell library was prepared and sequenced using a single-molecule real-time (SMRT) cell in a PacBio RS II sequencer at the McGill University and Génome Québec Innovation Centre (Canada) for long-read sequencing. This generated 95,374 raw subreads with an average length of 11,917 bp (total, 2.1 Gbp). PacBio data were assembled using the Hierarchical Genome Assembly Process (HGAP) pipeline v.2015 (4) to generate five contigs with an *N*_50_ value of 4,042,216 bp. Two short contigs were identified as erroneous due to homology with the longer contigs and were discarded. Overlapping contig ends indicated that the contigs were circular and were trimmed. Methylation motifs of GAYNNNNNCTTGY (type I gamma, 96.8% detected) and GTWWAC (type II beta, 95.3% detected) were identified and submitted to REBASE (5) for analysis.

Illumina sequencing libraries were prepared by DeepSeq (University of Nottingham, Nottingham, UK) using the Kapa HyperPlus kit (catalog number KK8510; Roche) and the Kapa single-indexed adapter kit, Illumina platforms, set B (catalog number KK8702; Roche). Genomic DNA was fragmented for 10 min, and 2 cycles of PCR were used during the library amplification stage. Postamplification solid-phase reversible immobilization (SPRI)-based size selection was performed using both 0.7 and 0.9 ratios of AMPure XP beads (catalog number A63882; Beckman Coulter) to DNA. Libraries were pooled in equimolar amounts, and final library quantification was performed using the Kapa library quantification kit for Illumina (catalog number KK4824; Roche). The library pool was sequenced on an Illumina NextSeq 500 instrument (NextSeq Control Software v.2.2.0) using a NextSeq 500 midoutput 300-cycle kit v.2.5 (catalog number 20024911; Illumina) to generate 4,204,264 150-bp paired-end reads totaling 782.3 Mbp. The reads passed standard Illumina quality control (QC) metrics and were trimmed and mapped to the PacBio unitigs using CLC Genomics Workbench v.12.0, with the default parameters. A total of 4,182,427 reads (99.48%) mapped to the unitigs to give two chromosomes (4,049,965 bp and 2,912,457 bp) and a megaplasmid (452,139 bp); in total, there was 7,414,561 bp with a GC content of 66.36% and 84× coverage. The sequence was annotated using the NCBI Prokaryotic Genome Annotation Pipeline. (6). The sequence has a symmetrical identity of 99.9738% to the Cupriavidus necator H16 sequence described by Pohlmann et al. (1), with a net increase of 16 protein-coding genes (6,543 total), 39 pseudogenes (263 total), and 3 tRNA genes (64 total) annotated. This represents an improvement in the accuracy of the genome sequence, achieved by using updated sequencing technology. This will improve the accuracy of both metabolic modeling and metabolic engineering in this industrially important strain.

### Data availability.

This sequence has been deposited with the NCBI under GenBank accession numbers CP039287, CP039288, and CP039289. The raw reads are available in the NCBI SRA under accession numbers SRX5785271 and SRX5785270 (BioProject number PRJNA531660).
